# Augmenting glycosylation‐directed folding pathways enhances the fidelity of HIV Env immunogen production in plants

**DOI:** 10.1002/bit.28169

**Published:** 2022-07-19

**Authors:** Emmanuel Margolin, Joel D. Allen, Matthew Verbeek, Ros Chapman, Ann Meyers, Michiel van Diepen, Phindile Ximba, Thopisang Motlou, Penny L. Moore, Jeremy Woodward, Richard Strasser, Max Crispin, Anna‐Lise Williamson, Edward P. Rybicki

**Affiliations:** ^1^ Department of Pathology, Division of Medical Virology, Faculty of Health Sciences University of Cape Town Cape Town South Africa; ^2^ Wellcome Trust Centre for Infectious Disease Research in Africa University of Cape Town Cape Town South Africa; ^3^ Institute of Infectious Disease and Molecular Medicine, Faculty of Health Sciences University of Cape Town Cape Town South Africa; ^4^ Biopharming Research Unit, Department of Molecular and Cell Biology University of Cape Town Cape Town South Africa; ^5^ School of Biological Sciences University of Southampton Southampton UK; ^6^ National Institute for Communicable Diseases of the National Health Laboratory Service Centre for HIV and STIs Johannesburg South Africa; ^7^ MRC Antibody Immunity Research Unit, Faculty of Health Sciences University of the Witwatersrand Johannesburg South Africa; ^8^ Centre for the AIDS Programme of Research in South Africa (CAPRISA) University of KwaZulu‐Natal, Congella Durban South Africa; ^9^ Department of Integrative Biomedical Sciences University of Cape Town Cape Town South Africa; ^10^ Department of Applied Genetics and Cell Biology University of Natural Resources and Life Sciences Vienna Austria

**Keywords:** chaperones, glyco‐engineering, glycosylation, immunogenicity, neutralization, occupancy, vaccine

## Abstract

Heterologous glycoprotein production relies on host glycosylation‐dependent folding. When the biosynthetic machinery differs from the usual expression host, there is scope to remodel the assembly pathway to enhance glycoprotein production. Here we explore the integration of chaperone coexpression with glyco‐engineering to improve the production of a model HIV‐1 envelope antigen. Calreticulin was coexpressed to support protein folding together with *Leishmania major* STT3D oligosaccharyltransferase, to improve glycan occupancy, RNA interference to suppress the formation of truncated glycans, and *Nicotiana benthamiana* plants lacking α1,3‐fucosyltransferase and β1,2‐xylosyltransferase was used as an expression host to prevent plant‐specific complex *N*‐glycans forming. This approach reduced the formation of undesired aggregates, which predominated in the absence of glyco‐engineering. The resulting antigen also exhibited increased glycan occupancy, albeit to a slightly lower level than the equivalent mammalian cell‐produced protein. The antigen was decorated almost exclusively with oligomannose glycans, which were less processed compared with the mammalian protein. Immunized rabbits developed comparable immune responses to the plant‐produced and mammalian cell‐derived antigens, including the induction of autologous neutralizing antibodies when the proteins were used to boost DNA and modified vaccinia Ankara virus‐vectored vaccines. This study demonstrates that engineering glycosylation‐directed folding offers a promising route to enhance the production of complex viral glycoproteins in plants.

## 1 INTRODUCTION

Molecular farming has long promised the production of low‐cost vaccines on a large scale (Rybicki, 2009) and the potential for rapid production time frames to respond to emerging outbreaks (Margolin et al., 2018). In reality, however, the platform has mostly been confined to niche applications (Stoger et al., 2014) and, with the exception of a few products that have undergone clinical evaluation, the development of many potentially valuable plant‐made proteins (PMPs) has not progressed beyond academia (E. A. Margolin et al., 2020). Although this is partly attributable to limited availability of good manufacturing practice (GMP)‐compliant production facilities (Stoger et al., 2014), a major obstacle for the widespread acceptance of plant molecular farming is the observation that the host cellular machinery does not always support the required posttranslational modifications that are critical for protein folding and functionality (E. A. Margolin et al., 2020). These constraints are particularly problematic for viral glycoproteins, which are important targets for vaccination (Sanders & Moore, 2021) and which often have unusually complex maturation requirements (Watanabe et al., 2019).

Consequently, efforts are increasingly aimed at addressing bottlenecks in planta that impair the production and functionality of PMPs (Margolin, Crispin, et al., 2020; E. A. Margolin et al., 2020). The low yields reported for many viral glycoproteins in plants (Margolin et al., 2018) are probably partly related to the host chaperone machinery, as the coexpression of human chaperones (Protein origami™) has been demonstrated to substantially improve the accumulation of human viral glycoproteins in *Nicotiana benthamiana* (Meyers & Margolin, 2018; Margolin, Oh, et al., 2020; Margolin, Verbeek, et al., 2020; Shin et al., 2021). This approach has also been reported to alleviate the endoplasmic reticulum‐related stress response and accompanying pathology, which was observed following expression of a soluble HIV‐1 envelope (Env) gp140 antigen (Margolin et al., 2019; Margolin, Oh, et al., 2020). The absence of furin proteases similarly complicates the production of properly mature viral glycoproteins, as has been described for other PMPs that require furin‐mediated processing for activation (Mamedov et al., 2019; Wilbers et al., 2016). This can be addressed by the coexpression of furin (Margolin, Oh, et al., 2020; Margolin et al., 2022), or by the use of cleavage‐independent antigen designs (Meyers et al., 2019; Margolin et al., 2019) where a flexible linker can compensate for proteolytic processing (Georgiev et al., 2015; Sarkar et al., 2018). Similar approaches could probably also be applied to other viral glycoproteins if the respective protease required for processing is absent (Margolin et al., 2020), and this approach is also compatible with the coexpression of human chaperones if both are found to be necessary for production of a given protein (Margolin, Oh, et al., 2020; Margolin et al., 2022).

The impact of the plant glycosylation machinery on viral glycoprotein production and immunogenicity is poorly understood, owing in part to the challenge of producing sufficient recombinant material for analysis and the need for direct comparison of plant‐derived material with the equivalent protein when produced in mammalian cells. Nonetheless, the glycosylation of viral glycoproteins is central to the folding of these proteins and host‐derived glycans direct the interaction of glycoproteins with various folding partners, co‐ordinate their trafficking along the secretory pathway and impose quality control to remove aberrantly folded proteins (Xu & Ng, 2015). PMPs are typically decorated with plant‐specific complex glycans that contain β1,2‐xylose and core α1,3‐fucose moieties (Montero‐Morales & Steinkellner, 2018). Truncated glycans and Lewis A structures are also commonly reported for plant‐produced proteins (Montero‐Morales & Steinkellner, 2018). Recently, a growing number of reports have established that some PMPs may be under‐glycosylated (Castilho et al., 2018; Goritzer et al., 2020; Singh et al., 2020) and, although this is a common limitation of heterologous expression systems, it appears to be exaggerated in plants, further complicating the widespread use of the production platform (Margolin, Crispin, et al., 2020; Singh et al., 2020).

The impact of plant‐specific glycosylation has been the subject of much debate, but it is important to distinguish between glycosylation patterns that merely reflect the machinery of the host cells and those that are of significance. The longest standing concern for PMPs is the potential of plant‐specific glycans to be immunogenic in humans (Gomord et al., 2010). Given that these epitopes are foreign, it has been suggested that immune responses against glyco‐epitopes could result in rapid clearance of the recombinant protein, or even anaphylaxis in extreme cases (Gomord et al., 2010). Concerns for the latter are reinforced by reports of hypersensitivity following the clinical administration of mammalian cell‐produced recombinant monoclonal antibodies containing foreign glyco‐epitopes. This is exemplified by the observation that anaphylactic reactions to the monoclonal antibody (mAb) cetuximab were associated with IgE responses to non‐native Galactose‐α−1,3‐Galactose, which arose following expression in the mouse SP2/0 cell line (Arnold & Misbah, 2008). Conversely, plant‐produced influenza virus‐like particles (VLPs) decorated with typical plant‐derived *N*‐glycans were safe in immunized volunteers with pre‐existing plant allergies, although transient induction of IgG and IgE to plant glyco‐epitopes was observed (Ward et al., 2014). More recently, plant‐produced severe acute respiratory syndrome coronavirus 2 (SARS‐CoV‐2) and rotavirus VLPs bearing their respective glycoproteins were similarly shown to be safe in humans (Kurokawa et al., 2021; Ward et al., 2021). The difference between vaccines and mAbs could be due to the very different dosing, with vaccines typically being used at far lower concentration and total amounts than mAbs. These observations have resulted in considerable optimism for molecular farming, which have been bolstered by reports of Medicago Inc.'s influenza and SARS‐CoV‐2 vaccines demonstrating efficacy and safety in phase 3 trials (Hager et al., 2022; Medicago, 2021; Ward et al., 2021). Their rotavirus VLP vaccine also generated promising immune responses in humans in an early stage clinical trial (Kurokawa et al., 2021). Nonetheless, many other viral glycoproteins may require more faithful recapitulation of their native glycosylation to support appropriate folding and immunogenicity (Margolin et al., 2021; Margolin, Crispin, et al., 2020).

Considerable effort has been devoted to “humanizing” the *N*‐linked glycosylation of PMPs by eliminating plant‐specific glycan moieties and by generating mammalian‐type *N*‐glycan extensions, which would not otherwise occur in plants (Montero‐Morales & Steinkellner, 2018). Notable achievements include the generation of mutant plants that are deficient for β1,2‐xylosyltransferase and α1,3‐fucosyltransferase expression (termed ∆XF), thereby eliminating the formation of plant‐specific complex glycans (Strasser et al., 2008), the suppression of endogenous hexosaminidases (HEXOs), to prevent the formation of truncated glycans (Shin et al., 2017), and increased glycan occupancy following the coexpression of *Leishmania major* STT3D oligosaccharyltransferase (LmSTT3D; Castilho et al., 2018; Goritzer et al., 2020). Success has also been reported for the production of PMPs containing mammalian‐type glycan extensions that are commonly observed on viral glycoproteins (Watanabe et al., 2019) including core α1,6‐fucosylation (Castilho et al., 2011), β1,4‐galactosylation (Schneider et al., 2015), and even sialylation, although these have not yet been applied to plant‐produced viral glycoproteins (Castilho et al., 2010). The development of these approaches presents a powerful toolbox to produce PMPs with tailor‐made glycosylation and offers the prospect of producing viral glycoproteins that recapitulate important features of their native glycosylation. Nonetheless, these approaches have not been well‐explored for viral glycoproteins and it is presently unclear how well they will work in the context of such heavily‐glycosylated targets (Margolin, Crispin, et al., 2020).

We have pursued plant‐based production of the HIV Env as a model viral antigen with complex folding and glycosylation requirements to explore how the plant cellular machinery impacts the production of such complex glycoproteins (Margolin et al., 2019). Env is highly dependent on the host cell machinery for its biosynthesis (Checkley et al., 2011) and therefore this presents an opportunity to investigate potential constraints along the secretory pathway that could impede the production of other similarly complex viral glycoproteins. The native protein is comprised of an extensively glycosylated trimer where approximately half the mass is attributed to host‐derived glycans (Stewart‐Jones et al., 2016). These glycans are critical for protein folding as they dictate interactions with the host chaperone machinery (Watanabe et al., 2019). They are also important for viral fitness where they protect vulnerable epitopes from neutralizing antibodies (Wei et al., 2003). The glycans decorating Env are predominantly oligomannose‐type as their dense clustering and the quaternary structure of the trimer sterically hinders access by host mannosidases, thereby impeding their maturation (Pritchard et al., 2015). More extensively processed, complex glycans are present at the base of the glycoprotein where they can be accessed by the host glycan processing machinery (Struwe et al., 2018). Although glycans can be recognized as part of epitopes targeted by neutralizing antibodies (Wibmer et al., 2016), holes in the glycan shield are more common targets for neutralization (Charles et al., 2021; McCoy et al., 2016; Pejchal et al., 2011; Yang et al., 2020) as protein epitopes are comparatively more immunogenic than host‐derived carbohydrates (Zhou et al., 2017). Accordingly, recombinant immunogens with artificial holes in the glycan shield or misfolded antigens, which expose regions that are sequestered in the virion‐associated trimer induce functionally irrelevant antibodies against these regions. Recapitulating the glycan shield is therefore often an important consideration for vaccine design and investigating plant‐based production of the HIV Env glycoprotein provides an opportunity to evaluate how closely the system can reproduce these features where necessary.

To establish the impact of plant‐specific glycosylation on viral glycoprotein production, we previously defined the site‐specific occupancy of several prototype antigens and compared them to the equivalent human embryonic kidney 293 cells (HEK293) cell‐produced proteins (Margolin et al., 2021). Notably, the plant‐produced material displayed increased oligomannose and truncated glycans, negligible complex‐type glycans, as well as elevated levels of under occupancy compared with the equivalent mammalian cell‐produced protein. The latter observation could account for the aggregation of recombinant HIV‐1 Env gp140 (Margolin et al., 2019) and Marburg virus glycoprotein (Margolin et al., 2021), which we previously observed following their expression in plants, as well as potentially impaired folding and immunogenicity of the gp140 immunogen (Margolin, Crispin, et al., 2020). In this current study, we sought to address these constraints using HIV Env gp140 as a challenging model antigen exhibiting extensive posttranslational modifications (Meyers et al., 2019; Margolin et al., 2019), with the intention of integrating approaches to support both chaperone‐mediated folding and glycosylation. Remodeling the secretory pathway presents a promising route to enhancing the fidelity of recombinant HIV and other complex viral glycoproteins in plants.

## 2 RESULTS

### 2.1 Production of a “glycan‐enhanced” HIV Env gp140 antigen in plants

Site‐specific glycan analysis of plant‐produced gp140 has previously shown that the antigen was under‐glycosylated in plants and that the protein contained truncated glycans that were absent in the equivalent mammalian cell‐produced protein (Margolin et al., 2021). Accordingly, in this study an integrated series of expression approaches (NXS/T Generation™) were conceived to address these constraints to produce an improved variant of the antigen in plants which is subsequently referred to as “glycan‐enhanced” (GE) gp140. Here we use the phrase GE to reflect material generated in plants using these strategies, with the aim of improving the glycosylation so that it more closely resembles the equivalent mammalian cell‐produced protein. The protein was coexpressed with human calreticulin (CRT) as previously described, to improve production yields (Margolin, Oh, et al., 2020), *L. major* LmSTT3D was coexpressed to improve glycan occupancy (Castilho et al., 2018), and an RNA interference construct was coexpressed to supress activity of HEXO3 (HEXO3RNAi), which is responsible for truncating glycans to yield paucimannosidic structures and eventually core structures (Shin et al., 2017). These expression constructs were validated in previous studies and their effect has already been reported (Castilho et al., 2018; Margolin, Oh, et al., 2020; Margolin et al., 2022; Shin et al., 2017) These approaches were combined in *N. benthamiana* ∆XF to prevent the formation of undesired species‐specific complex glycans (Strasser et al., 2008), with the intention to produce an antigen with improved glycosylation but lacking undesirable plant‐specific glycan modifications (Strasser et al., 2008). The antigen was also produced in wild‐type (WT) *N. benthamiana* by CRT coexpression (Margolin et al., 2021; Margolin, Oh, et al., 2020) and in stably transfected HEK293 cells (van Diepen et al., 2019), as previously described, for comparison (Table 1).

**Table 1:** Summary of expression approaches implemented to produce recombinant HIV Env gp140 in plants and mammalian cells

Antigen name	Expression host	Accessory proteins	Desired impact
WT	*N. benthamiana*	CRT	↑Yield
GE	*N. benthamiana* ∆XF	CRT	↑Yield
		LmSTT3D	↑*N*‐glycan occupancy
		HEXO3RNAi	↓Truncated N‐glycans
HEK293	HEK293 cells	NA	NA

Abbreviations: ∆XF, plants lacking α1,3‐fucosyltransferase and β1,2‐xylosyltransferase; CRT, calreticulin; HEK293, human embryonic kidney 293 cells; HEXO3RNAi, RNA interference to suppress Hexosaminidase 3; LmSTT3D, *Leishmania major* STT3D oligosaccharyltransferase; NA, not applicable; WT, wild type.

**Figure 1:**
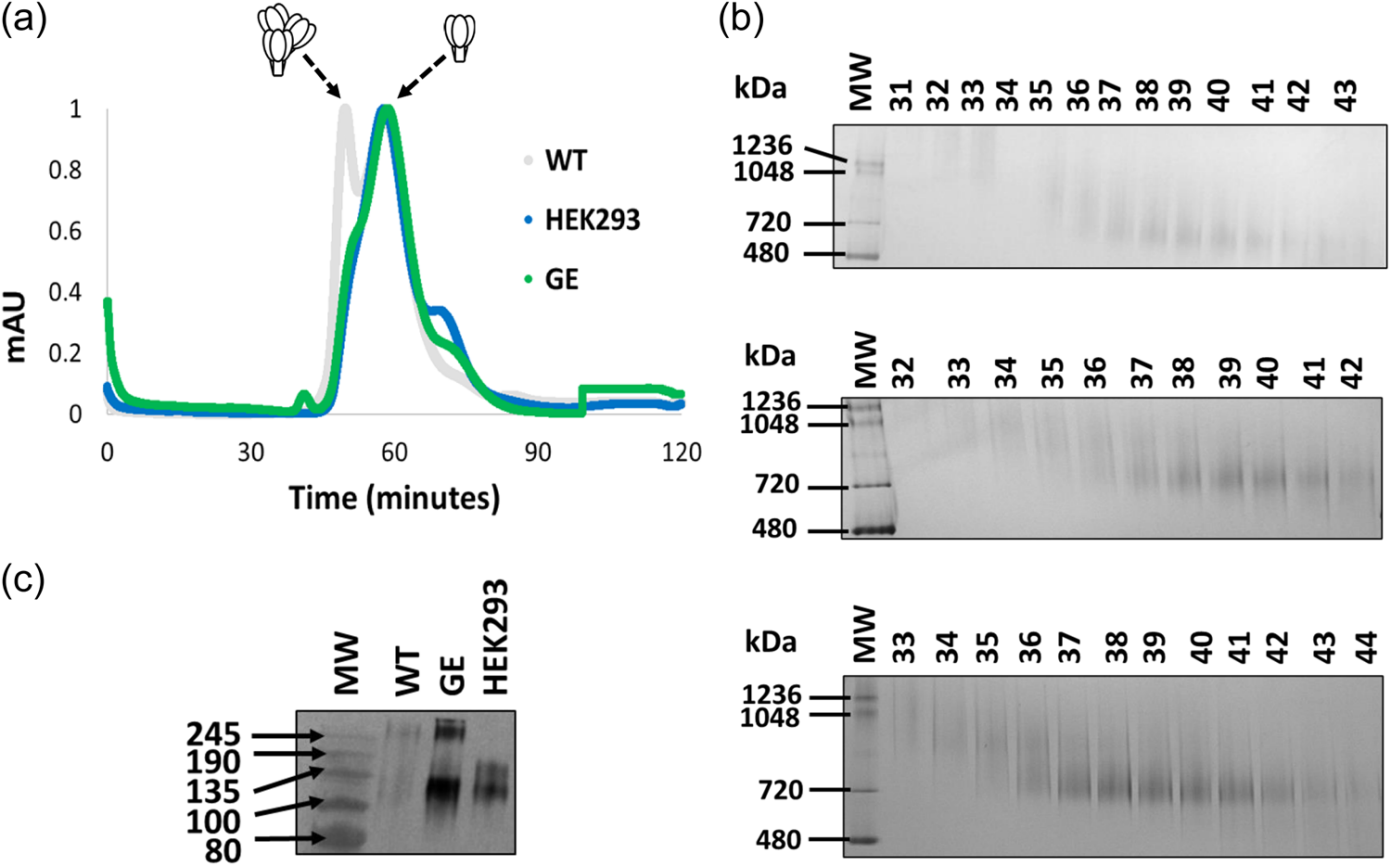
Production of HIV envelope (Env) gp140 in plants with improved glycosylation. (a) Overlayed normalized size exclusion chromatography elution profiles of gp140 when produced in human embryonic kidney 293 (HEK293) cells or in plants with modified (glycan‐enhanced [GE]) or unmodified glycosylation (wild type [WT]). The peaks corresponding to aggregates and putative trimers are indicated by the cartoon image above the figure. (b) Coomassie‐stained blue native polyacrylamide gel electrophoresis (BN‐PAGE) gel comprising of size exclusion chromatography (SEC)‐fractionated material from all three protein variants. The number above each lane refer to a corresponding fraction from size exclusion chromatography. Top: WT; middle: GE; bottom: HEK293. (c) Western blot analysis of the pooled fractions following SEC. HIV Env was detected using polyclonal goat anti‐gp120 antibody.

The three variants of the protein were purified by sequential *Galanthus nivalis* lectin affinity chromatography and gel filtration steps that were developed during previous studies (Margolin et al., 2019; van Diepen et al., 2019). The overlaid size exclusion chromatography profiles showed a marked improvement in production of the GE gp140 compared with the WT antigen: this exhibited a marked shift to the left of the elution profile that was consistent with increased aggregation, as previously observed (Margolin et al., 2021; Figure 1a). The overlaid gel filtration profiles of the GE gp140 and mammalian cell‐produced proteins were almost perfectly aligned, with the predominant species consistent with reports for trimeric HIV Env gp140 (van Diepen et al., 2019). The GE gp140 and mammalian cell‐produced proteins both displayed profiles reminiscent of trimeric material, and lacked the extensive aggregate peak observed for the WT plant protein. Resolution of the gp140 variants on Coomassie‐stained blue native polyacrylamide gel electrophoresis (BN‐PAGE) gels largely mirrored these observations, although the ability to discriminate between putative trimers and aggregates was markedly decreased owing in part to lower yields for the WT protein (Figure 1b). These data suggest that the host N‐linked glycosylation machinery does not support efficient glycosylation of the gp140 and that inefficient glycosylation is responsible for undesired aggregation of the protein in plants.

Western blot analysis of the purified proteins also demonstrated a considerable improvement in the production of the antigen in plants following engineering (Figure 1c). The WT protein was poorly resolved by SDS‐PAGE and presented as a diffuse smear, as previously described (Margolin et al., 2019). In contrast, the GE antigen yielded a defined band, although some higher order products were also observed which likely represent a minor population of misfolded protein. The GE protein was slightly smaller than the equivalent mammalian cell‐produced protein, as is expected given the lack of complex N‐linked glycans in *N. benthamiana* ∆XF, which would be imparted by mammalian cells.

### 2.2 Site‐specific glycosylation of GE gp140

**Figure 2:**
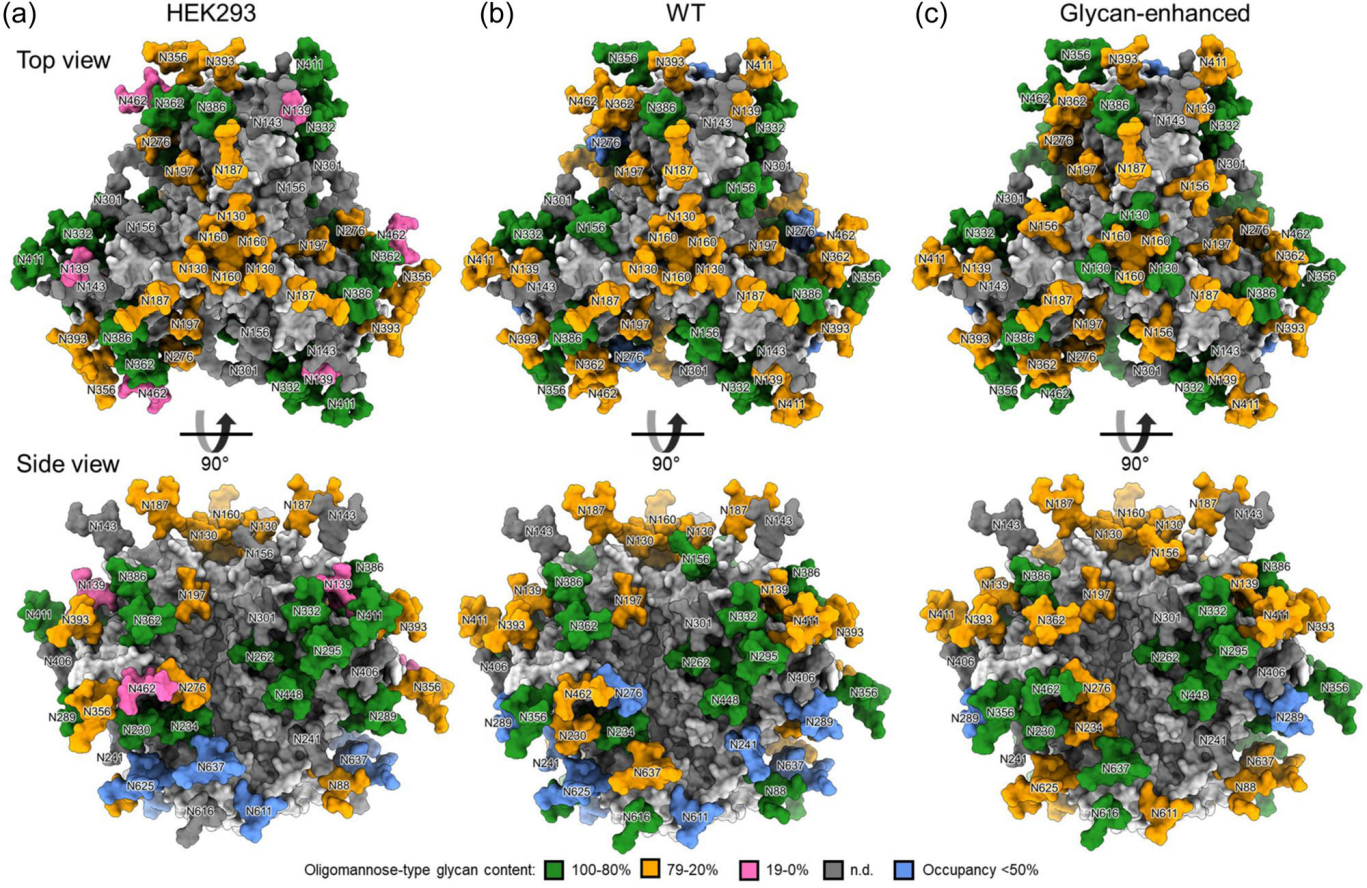
Site‐specific glycosylation of CAP256 gp140 produced in mammalian cells and in plants with either unmodified or modified glycosylation. (a) Heat map depicting the abundance of oligomannose‐type glycans when HIV envelope (Env) gp140 is produced in human embryonic kidney 293 (HEK293) mammalian cells. The model was constructed using SWISS‐MODEL using the structure of BG505 NFL as a template (6B0N). A representative glycan corresponding to the most abundant glycoform present at each site was modeled and sites are colored according to the abundance of oligomannose‐type glycans detected at each site. All glycans are labeled according to the asparagine they are attached to, following alignment with HxB2. Where an *N*‐linked glycan site was occupied by *N*‐linked glycans on <50% of sites, the glycan modeled was colored blue. (b) The model generated for HEK293 produced CAP256 Env was recolored according to the site‐specific glycan data for WT material. (c) The model generated for HEK293 produced CAP256 Env was recolored according to the site‐specific glycan data for “glycan‐enhanced” Env.

To verify the impact of our integrated engineering approach, the quantitative site‐specific glycosylation of the three gp140 variants was determined by liquid chromatography‐mass spectrometry. Modeling of the site‐specific glycosylation data onto a model of the trimer enables the distribution of the various glycans on the trimer to be visualized more easily (Figure 2). This representation also depicts the location of large holes in the glycosylation shield, which are likely targets of antibodies given the observation that protein epitopes are comparatively more immunogenic than host‐derived glycosylation (Zhou et al., 2017). As expected, lower levels of oligomannose‐type glycans are visible on the mammalian protein where the glycosylation is more sparse and the glycans are more accessible for processing by host mannosidases (Figure 2a). This representation of the glycosylation also reveals that although the GE protein is less glycosylated than the mammalian cell protein at several sites (Supporting Information: Tables S1–S3), engineering has resulted in only a single site with <50% under occupancy (N289; Figure 2c). This contrasts to the mammalian cell‐produced protein where N625, N637, and N611 are all under occupied in >50% of the sites sampled (Figure 2a). This is similarly observed in the WT protein for N625 and N611 which, in addition to these sites, also exhibits <50% occupancy at several other potential *N*‐linked glycosylation sites (PNGs) (N241, N289, N276; Figure 2b).

**Figure 3:**
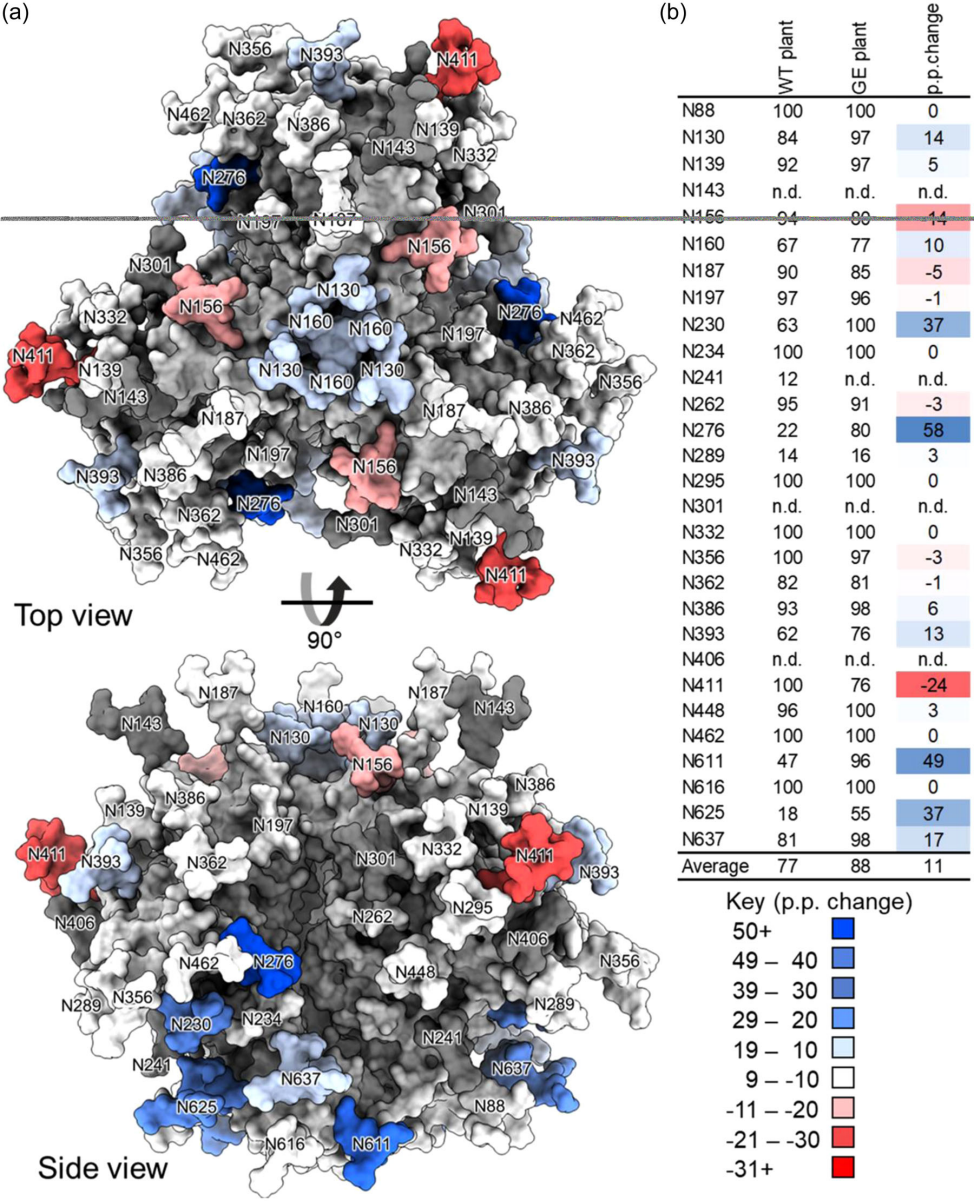
Comparison of the glycan occupancy of recombinant HIV envelope (Env) gp140 when produced in *N. benthamiana* using integrated host and transient glyco‐engineering (“glycan‐enhanced”) or no glyco‐engineering expression approaches (wild type [WT]). (a) The percentage point changes in glycan occupancy are labeled onto the model generated in Figure 2. A positive change represents a glycan site that is more occupied on the “glycan‐enhanced” material compared with the WT. (b) Site‐specific glycan occupancy of recombinant HIV Env gp140 produced in WT and “glycan‐enhanced” *N. benthamiana*. The change in glycan occupancy is colored, with red representing a decrease and blue an increase.

**Figure 4:**
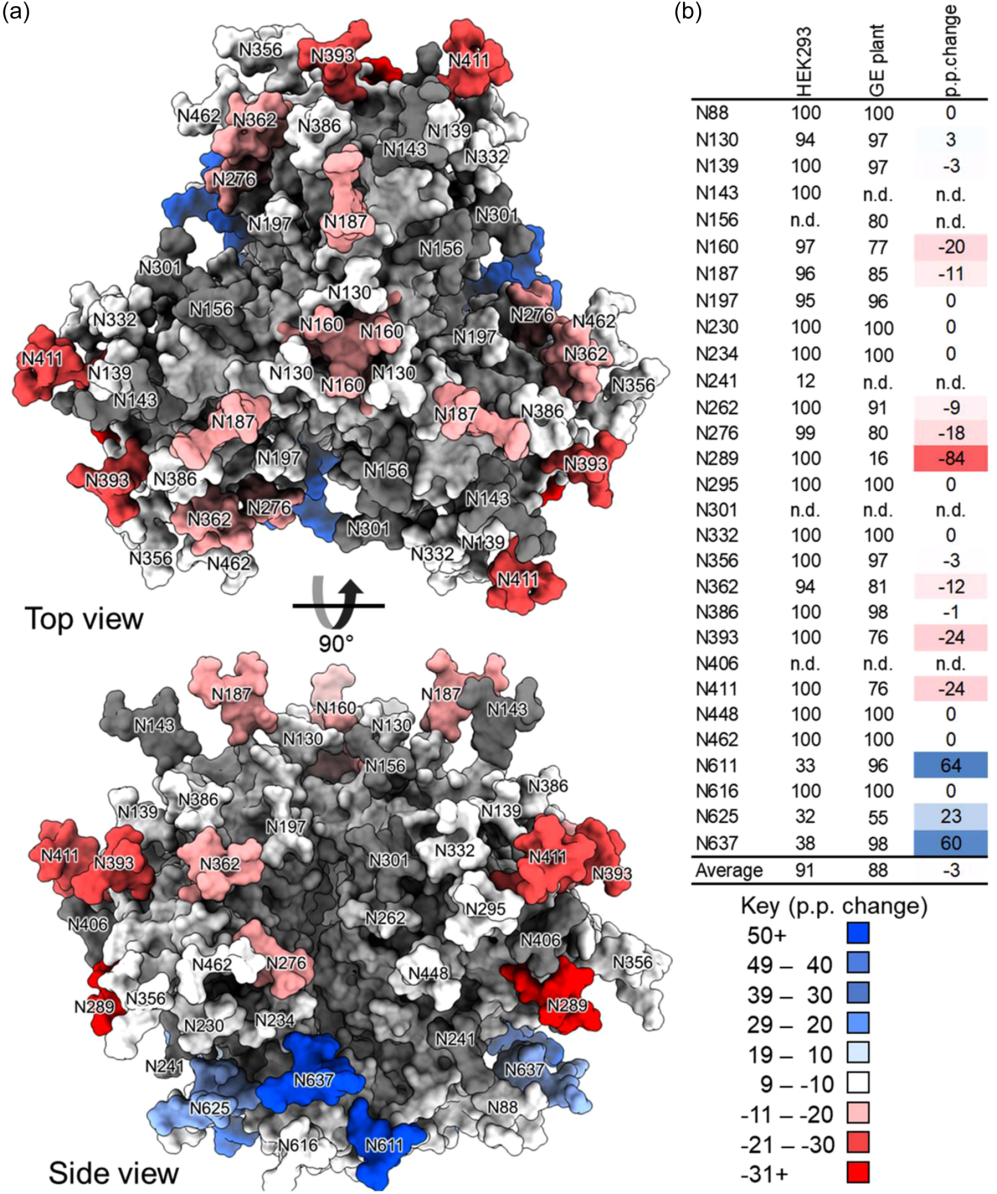
Comparison of the glycan occupancy of recombinant HIV envelope (Env) gp140 when produced in *N. benthamiana* using integrated host and transient glyco‐engineering (“glycan‐enhanced”) or mammalian cells (HEK293). (a) The percentage point changes in glycan occupancy are labeled onto the model generated in Figure 2. A positive change represents a glycan site that is more occupied on the “glycan‐enhanced” material compared with the HEK293‐derived material. (b) Site‐specific glycan occupancy of recombinant HIV Env gp140 produced in WT and “glycan‐enhanced” *N. benthamiana*. The change in glycan occupancy is colored, with red representing a decrease and blue an increase.

To further explore changes in glycosylation between the three proteins, the site‐specific change in glycan occupancy (Figures 3 and 4) and glycan compositions (Supporting Information: Tables S1–S3) were compared for the GE and the WT plant protein (Figure 3 and Supporting Information: Table S4) and the GE gp140 and the mammalian cell‐produced protein, respectively (Figure 4 and Supporting Information: Table S5). The changes in glycosylation are presented as the percentage point change at each sequon, indicating a relative increase or decrease for the proteins being compared. These data are informative of differences in glycosylation at specific PNGs, which can indicate whether the recombinant protein is appropriately folded (Behrens et al., 2017) or if key glycans comprising epitopes targeted by broadly neutralizing antibodies are present (Behrens & Crispin, 2017). The site‐specific composition of each of the individual proteins is reflected in Supporting Information: Tables S1–S3.

The data in Figure 3 demonstrate a marked decrease in unoccupied PNGs at multiple sites across the GE protein compared with the WT antigen (N130, N139, N160, N230, N289, N386, N393, N448, N611, N625, N637). The observed increase at N160 is encouraging as this glycan forms part of an epitope at the trimer apex that is frequently targeted by broadly neutralizing antibodies (Wibmer et al., 2015). A slight increase in glycan occupancy is similarly observed for N386 which forms part of the intrinsic mannose patch (Behrens & Crispin, 2017). Oligomannose glycans predominate at all PNGs in both the GE and WT proteins (Supporting Information: Tables S1 and S2). Notable differences are observed in the mannose processing states at several PNGs. In many instances, the GE protein contains elevated levels of M5 glycans compared with the WT, such as N234 (Supporting Information: Table S4). These arise through the action of Golgi‐resident mannosidases which systematically trim away mannose moieties to yield a M5 intermediate for the formation of complex glycans. Lastly, on average the GE gp140 contains similar levels of truncated and core glycans suggesting that suppression of HEXO has a negligible effect (Supporting Information: Table S4).

Comparing the GE protein to the mammalian cell‐produced antigen (Figure 4) reveals that despite improvements, the GE antigen still contains lower occupancy at numerous sites (N160, N187, N262, N276, N289, N362, N386, 393, N411). This includes a key broadly neutralizing antibody epitope, the N160 glycan, at the trimer apex, which has been observed to form target epitopes during natural infection, including for the CAP256 virus, which was used to design the immunogens described in this study (Moore et al., 2011). Nonetheless, the GE protein contains improved glycan occupancy compared with the progenitor WT protein (Supporting Information: Tables S1, S2, and S4). Interestingly, four other PNGs in the GE protein exhibited increased occupancy compared with the mammalian protein (N130, N611, N625, N637). As expected, the mammalian protein also contains a diverse array of complex glycans which are lacking in the GE protein (Supporting Information: Table S5). Nonetheless, oligomannose‐type glycans predominate at N160, N332, and are abundant at N393 (Supporting Information: Table S3) as expected for the intrinsic mannose patch where glycan processing is sterically hindered (Behrens & Crispin, 2017). Mannose trimming also notably decreased in the GE protein compared with when the antigen was expressed in mammalian cells.

**Figure 5:**
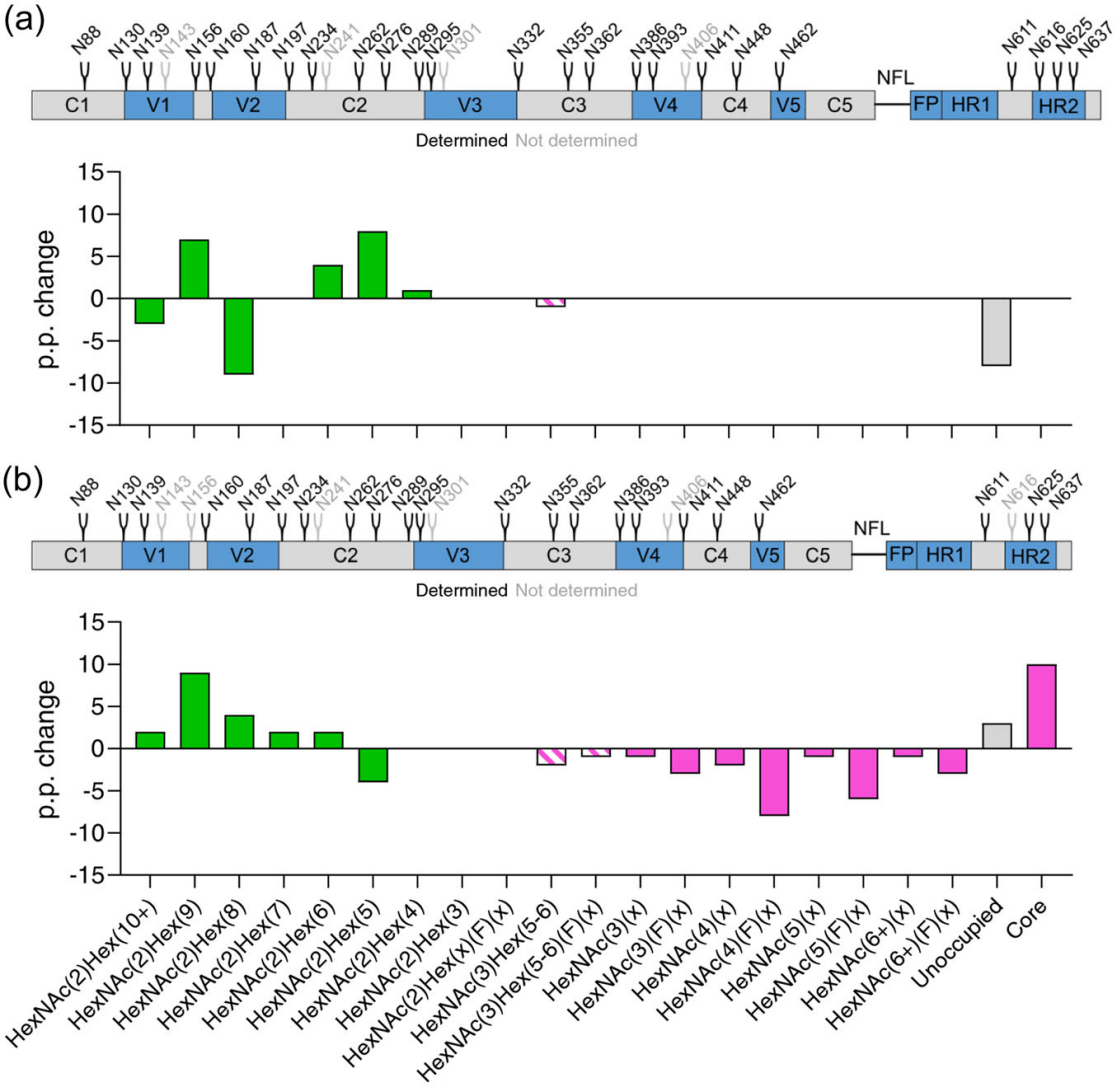
The average change in glycosylation across all detected sites on recombinant HIV envelope (Env) gp140 when produced in plants and mammalian cells. (a) Comparison of the glycosylation of “glycan‐enhanced” gp140 compared with the nonoptimized protein, both proteins are produced in *N. Benthamiana*. (b) Comparison of the glycosylation of the “glycan enhanced” gp140 compared with the mammalian cell‐derived protein. In both a and b, the glycosylation of the “glycan‐enhanced” protein is presented relative to the comparator protein and, therefore, positive and negative values indicate an increase or decrease in the abundance of a particular glycan species in the “glycan‐enhanced” antigen. Schematics above each graph display the HxB2 aligned CAP256 NFL *N*‐linked glycan sites, with sites labeled in black representing sites that were resolved by liquid chromatography‐mass spectrometry (LC‐MS) and gray the ones that were not.

An alternate approach to interpreting these data sets is to view them from a global perspective rather than analyzing them for each PNGs. This highlights the relative abundance of different glycan species across the entire protein to identify expression‐system dependent patterns that are less confounded by how well the protein is folded. It should be noted, however, that as all sites are not included in the data this should be considered as a representation of the overall trend of the data rather than as an absolute measure. A more accurate indication of the global changes between the proteins would require further delineation of the site‐specific glycan composition at every PNGs on each protein to avoid any confounding effect. The GE protein is compared with the WT protein in Figure 5a and then to the mammalian cell‐produced antigen in Figure 5b. As before, the glycan composition is depicted as the percentage point change in the GE protein compared with its comparator. Therefore, positive and negative values indicate an increase or decrease in the relative abundance of a particular glycan, respectively. It is important to note that certain sites could not be resolved, for example, N406 on all samples analyzed, and the values presented represent the average of sites that could be obtained. The GE gp140 also displays improved glycan processing as evidenced by decreased Man8 and elevated Man6 and Man5 glycans. The extent of under occupancy is also noticeably reduced in the GE protein.

However, when the GE gp140 is compared with the mammalian cell‐produced protein (Figure 5b), a slight increase in glycan compositions are observed that likely correspond to glucosylated Man9 structures (HexNAc(2)Hex(10+). Although this may reflect slightly less efficient folding in plants, this is equally likely to arise from differences in purification methods for the two systems. As observed with the site‐specific analysis, the GE protein contains almost exclusively oligomannose‐type glycans with considerably less processing than in mammalian cells where a considerable amount of complex glycans are observed.

The data from all three protein variants are summarized in Figure 5 and Supporting Information: Tables S1–S3, which quantitatively reflects differences in their glycosylation. Although oligomannose glycans predominate in mammalian cells (57%), this is notably increased in plants with the GE protein containing 71% oligomannose glycans compared with 66% in the WT protein. The mammalian cell‐produced protein further contains 24% complex type glycans, 20% fucosylation, and 3% neuraminic acid, where these are negligible or entirely absent in both plant‐produced proteins. The quantitative summary of this data highlights the extent of under‐glycosylation in the WT antigen where 23% of sites are not occupied. The GE protein is substantially improved with only 12% of sites being unoccupied, compared with 9% in the mammalian protein. Although there is only a 3% point difference in glycosylation between the GE protein and the mammalian‐cell derived protein, this is still a difference with important implications for protein folding and immunogenicity (Margolin, Crispin, et al., 2020). Finally, it is important to note that although the proteins were captured using *G. nivalis* lectin, which specifically binds high mannose carbohydrates, it has been shown that this does not unduly bias the glycosylation analysis of HIV Env proteins purified with this resin (Pritchard et al., 2015).

### 2.3 Characterization of GE gp140

Based on the aberrant folding, poor glycosylation and difficulties in producing a homogenous batch of the WT antigen, this was not pursued further. All data generated thus far demonstrated a considerable improvement for the GE protein. Consequently, all subsequent work in this study focused on comparing this improved antigen to the mammalian cell‐produced protein.

**Figure 6:**
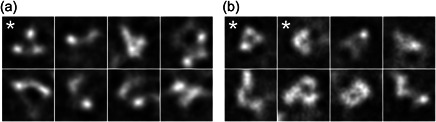
Estimating the proportion of correctly assembled trimeric gp140. (a) Sample of two‐dimensional (2D) classes of negatively stained gp140 expressed in HEK293 cells. The second most populous class (*) contains 5% of the particles (355/7158) and shows approximately the correct size and 3‐fold symmetry to be identified as trimeric gp140. (b) Sample of 2D classes of gp140 expressed in plants with modified glycosylation (“glycan‐enhanced”). Two classes (*) 6% of the images (1263/30444) show ~3‐fold symmetry and could represent correctly assembled gp140.

Although gel filtration is commonly used to purify oligomeric proteins it cannot discriminate between well‐ordered and aberrantly folded proteins (Ringe et al., 2013). The hallmark of a well‐folded trimer is regarded as a compact three‐lobed structure as viewed by negative‐stain electron microscopy (EM) and preferential reactivity with broadly neutralizing antibodies compared with nonneutralizing antibodies (Sanders et al., 2013). The purified GE and HEK293 cell‐produced antigens were therefore visualized by negative‐stain EM to investigate whether the antigens formed native‐like trimers and to compare the structures of the two proteins (Figure 6). Considerable heterogeneity was observed in both systems and only ~5% of the particles formed discrete particles with threefold symmetry and the correct size to be identified as gp140 timers. Nonetheless, the plant‐produced material appeared comparable to protein produced in mammalian cells based on this analysis. The data further suggest that the plant cellular machinery is capable of supporting appropriate oligomerization, which is an important consideration for the production enveloped viral glycoprotein immunogens.

### 2.4 Immunogenicity comparison of plant‐produced GE and mammalian cell‐produced gp140

**Figure 7:**
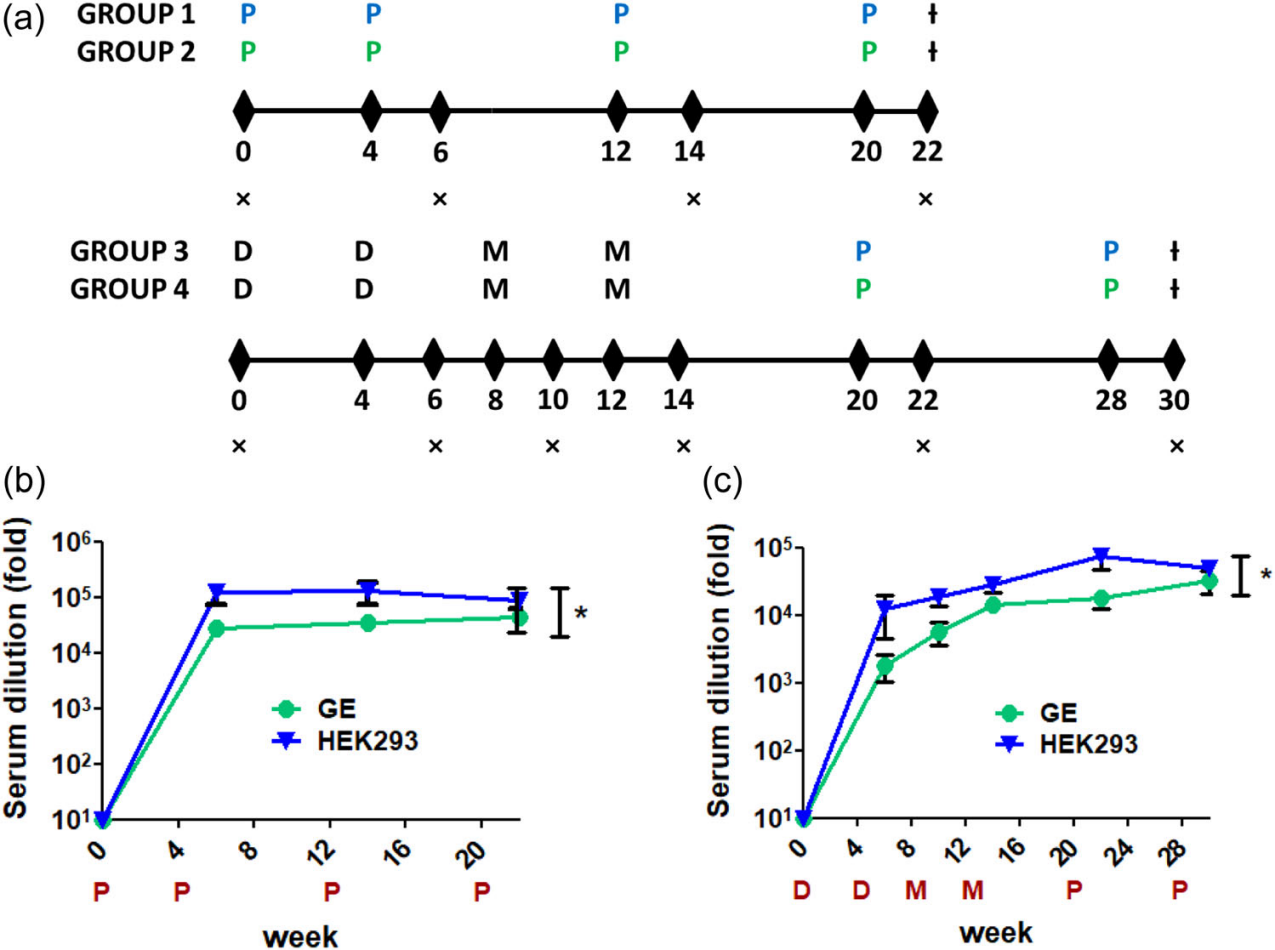
Immunogenicity comparison of “Glycan‐enhanced” and human embryonic kidney 293 (HEK293) cell‐produced HIV envelope (Env) gp140 in rabbits. (a) Schedule of immunizations and bleeds. Animals (*n* = 5) in Group 1 and 2 were immunized four times with purified “glycan‐enhanced” gp140 or gp140 from HEK293 cells. Animals in Groups 3 and 4 were primed with two DNA and two modified vaccinia ankara (MVA) vaccinations encoding mosaic Gag and CAP256 SU gp150, before boosting with purified protein. Animals in Group 3 were injected with the plant‐produced “glycan‐enhanced” antigen as a boost, whereas animals in Group 4 received the HEK293 cell‐derived protein as a boost. D,  DNA; M, MVA; ×, bleed, Ɨ, sacrifice; P, protein; P (green), glycan‐enhanced; P (blue), HEK293 cell‐derived). (b) Serum‐binding antibodies induced by the PPPP vaccination regimen. (c) Serum‐binding antibodies induced by the DDMMPP vaccination regimen.

The immunogenicity of the recombinant proteins was tested in rabbits, which are an accepted small animal model for investigating the induction of antibodies against subunit HIV Env vaccines (Sanders & Moore, 2017). This experiment was conceived to determine whether the host engineering approaches implemented in this study yielded an equivalent antigen to the mammalian cell‐produced vaccine. Accordingly, rabbits were immunized in two different vaccination regimens as reflected in Figure 7a. The first regimen directly compared the plant‐produced GE antigen to the mammalian cell‐derived protein with four immunizations of purified protein. The second regimen compared the proteins as a subunit boost with DNA and modified vaccinia ankara (MVA) primes encoding HIV‐1 subtype C mosaic Gag, which buds to form VLPs, and a membrane‐bound HIV‐1 CAP256 SU gp150 antigen, which decorates the surface of the particle (van Diepen et al., 2019). This gp150 antigen is matched to the subunit trimer expressed in this study and differs only by the presence of a transmembrane domain, which enables the antigen to be presented on the surface of coexpressed Gag (van Diepen et al., 2019). This vaccination regimen was previously reported to elicit autologous neutralizing antibodies in rabbits (Chapman et al., 2020; van Diepen et al., 2019).

Binding antibodies were quantified by enzyme‐linked immunosorbent assay (ELISA) using a 1:1 ratio of GE:HEK293 cell‐derived protein to eliminate any expression system‐dependent bias. Animals in both experiments developed high titers of binding antibodies, which were detectable at the first time point analyzed (Figure 7b,c). Binding antibody titers in rabbits immunized with the HEK293 cell‐derived protein in the PPPP experiment plateaued after the first immunization and appeared to decay slightly after the final protein immunization. In contrast, binding antibodies increased slightly over the time course in animals immunized with the GE antigen (Figure 7b). Animals immunized with the HEK293 cell‐produced protein exhibited a statistically significant trend towards increased binding antibody titers over the course of the experiment (two‐way analysis of variance [ANOVA], *p* < 0.05), although by the terminal bleed the titers had converged.

Both groups of animals in the DDMMPP experiment developed increasing titers of binding antibodies following each immunization, with the exception of the final time point in animals boosted with the HEK293 cell‐produced protein. Surprisingly, animals in the DDMMPP group receiving the HEK293 cell protein displayed a trend towards higher binding antibodies even after priming immunizations, which were identical in both groups. This was statistically significant (two‐way ANOVA, *p* > 0.05), although by the terminal bleed the titers were comparable. On closer interrogation of the data this trend could be attributed to 2/5 abnormally high responders to the DNA immunizations in the HEK293 cell protein boost group.

Serum samples from both groups were also assessed for neutralization against pseudotyped HIV Subtype C viruses, using the standardized assay that has been described previously (Margolin et al., 2019). Viruses were selected that represented a range of neutralization sensitivities: Tier 1 viruses are lab‐passaged isolates that are unusually sensitive to neutralization, whereas Tier 2 viruses are more neutralization resistant and represent circulating isolates (Seaman et al., 2010). Although the induction of antibodies against tier 2 viruses is highly desirable this has only become achievable in recent years due to advances in structure‐based immunogen design and different viral envelopes differ in their propensity to induce neutralizing antibodies (Sanders & Moore, 2017).

Both groups from the protein only experiment developed neutralizing antibodies against the Tier 1A (MW965) and Tier 1B (6644) pseudoviruses. However, no neutralization was observed for the vaccine‐matched Tier 2 virus (CAP256 SU) in this vaccination regimen (Table 2). Both antigens elicited similar responses against MW965 with median titers of 2698 and 2911 induced by the plant‐derived and mammalian cell‐produced proteins, respectively. Both proteins also elicited comparable titers against 6644 with median titers of 100 and 118 observed for the plant and HEK293 proteins, respectively. Low levels of background were observed against pseudotyped VSV‐G, a negative control, for two of the rabbits immunized with the plant‐produced protein, although these were not observed for any of the HIV‐Env pseudotyped viruses.

**Table 2:** Neutralizing antibody titers (ID_50_) in animals immunized with plant‐produced protein (Group 1) and HEK293 cell‐derived protein (Group 2)

		ID_50_							
		VSV‐G		MW965		6644		CAP256 SU	
Animal #	Group#	w0	w22	w0	w22	w0	w22	w0	w22
**RB1**	**1**	24	<20	<20	800	<20	27	<20	<20
**RB2**	**1**	<20	<20	<20	5965	<20	111	<20	<20
**RB3**	**1**	<20	<20	<20	5511	<20	67	<20	<20
**RB4**	**1**	<20	<20	<20	***2698***	<20	162	<20	<20
**RB5**	**1**	20	<20	<20	641	<20	***100***	<20	<20
**RB6**	**2**	<20	<20	<2	1672	<20	76	<20	<20
**RB7**	**2**	<20	<20	<20	2438	<20	***118***	<20	<20
**RB8**	**2**	<20	<20	<20	***2911***	25	50	<20	<20
**RB9**	**2**	<20	<20	<20	8573	<20	238	<20	<20
**RB10**	**2**	<20	<20	<20	3895	<20	282	<20	<20

*Note*: MW965 and 6644 represent Tier 1A and Tier 1B viruses, respectively, whereas CAP256 SU is the matched autologous virus. Week 0 comprises the pre‐bleed, whereas Week 22 is the terminal bleed. VSV‐G is the negative control. The median titers are indicated in italics and bold where all animals produced neutralizing antibodies against the pseudovirus of interest.

In the priming experiment, neutralizing antibodies were quantified after the final MVA (Week 14) and at the terminal endpoint (Week 30). Neutralizing antibodies were observed against both MW695 and 6644 at both Week 14 and Week 30 (Table 3). Autologous neutralizing antibodies were observed in a single animal at Week 14 and in 3/5 animals in both groups at Week 30. A single animal in the group boosted with the mammalian cell‐derived protein developed a high titer autologous response (>540) whereas the other responders were considerably lower (<100). Nonetheless, it is encouraging to observe similar response rates between the GE protein and the mammalian cell‐produced antigen. This also constitutes an incremental improvement compared with previous studies with the plant‐produced immunogen where no autologous neutralizing antibodies were observed (Margolin et al., 2019).

**Table 3:** Neutralizing antibody titers generated by the DDMMPP regimen investigating plant‐produced (Group 3) and HEK293 cell‐derived (Group 4) HIV Env gp140

		ID_50_											
		VSV‐G			MW965			6644			CAP256 SU		
Animal #	Group#	w0	w14	w30	w0	w14	w30	w0	w14	w30	w0	w14	w30
**RB1**	**3**	<20	<20	<20	<20	503	***434***	<20	69	30	<20	31	86
**RB2**	**3**	<20	<20	<20	<20	1748	919	46	126	57	23	<20	<20
**RB3**	**3**	<20	<20	<20	<20	***335***	115	<20	54	***31***	<20	<20	38
**RB4**	**3**	<20	<20	<20	<20	316	558	28	***74***	71	<20	<20	25
**RB5**	**3**	<20	<20	<20	<20	233	349	<20	88	23	<20	<20	<20
**RB6**	**4**	<20	<20	<20	<20	620	2147	21	68	107	<20	<20	>540
**RB7**	**4**	<20	<20	<20	<20	360	719	59	32	51	21	<20	40
**RB8**	**4**	<20	<20	<20	29	***183***	***1396***	<20	<20	56	<20	<20	<20
**RB9**	**4**	<20	<20	<20	<20	80	42	<20	<20	<20	<20	<20	<20
**RB10**	**4**	<20	<20	<20	<20	58	3116	25	29	172	<20	<20	33
		<20	20–99	100–499	500–999	>1000							

*Note*: Week 0 comprises the pre‐bleed, Week 14 comprises the bleed after the second modified vaccinia ankara immunization, and Week 30 is the terminal bleed. The median titers are indicated in italics and bold where all animals produced neutralizing antibodies against the pseudovirus of interest. MW965 and 6644 represent Tier 1A and Tier 1B viruses, respectively, whereas CAP256 SU is the matched autologous virus.

### 2.5 Experimental procedures

#### 2.5.1 Recombinant HIV Env gp140 production in plants

Transient protein expression in plants was conducted by agroinfiltration of *N. benthamiana* WT and *N. benthamiana* ∆XF plants (Strasser et al., 2008). Recombinant *Agrobacterium tumefaciens* AGL1 strains encoding CAP256 SU gp140 and human CRT have already been reported (Margolin et al., 2019; Margolin, Oh, et al., 2020). Previously described expression constructs for LmSTT3D (Castilho et al., 2018) and HEXO3RNAi (Shin et al., 2017) were transformed into *A. tumefaciens* GV3101:pMP90 for this study. The transient coexpression of multiple proteins in plants was achieved by agroinfiltrating a mixed suspension of *A. tumefaciens* strains, as previously described (Margolin, Oh, et al., 2020). The bacterial inoculum for each strain was adjusted to a final OD_600_ of 0.5 for all strains, except in the case of *A*. tumefaciens encoding LmSTT3D, which was infiltrated at a final OD_600_ of 0.25 to minimize the associated toxicity. Biomass from agroinfiltrated plants was harvested 4‐5 days post agroinfiltration and either processed immediately or stored at −80°C. Recombinant HIV Env gp140 was purified by sequential *G. nivalis* lectin (GNL) affinity chromatography and gel filtration procedures, as previously reported (Margolin et al., 2019).

#### 2.5.2 Recombinant HIV Env gp140 production in mammalian cells

Protein production in mammalian cells was conducted using a previously generated adherent human embryo kidney cells 293 (HEK293) stable cell line (van Diepen et al., 2018). Following propagation of the cells in the presence of serum, protein expression was initiated under serum‐free conditions and recombinant gp140 was purified by GNL and size exclusion chromatography, as previously described (van Diepen et al., 2019). The purified protein was quantified using the *DC* protein assay (BioRad).

#### 2.5.3 Gel filtration comparison of plant and mammalian cell‐produced HIV Env gp140

Gel filtration profiles for plant‐produced and mammalian cell‐derived HIV Env gp140 were normalized as previously described (Margolin et al., 2021) and overlayed for comparative purposes.

#### 2.5.4 Site‐specific *N*‐glycan analysis of recombinant HIV Env gp140

Aliquots of Env produced in WT *N. benthamiana*, GE *N. benthamiana* and HEK293 cells were denatured for 1 h in 50 mM Tris/HCl, pH 8.0 containing 6 M of urea and 5 mM dithiothreitol (DTT). Next, Env proteins were reduced and alkylated by adding 20 mM iodoacetamide (IAA) and incubated for 1 h in the dark, followed by a 1 h incubation with 20 mM DTT to eliminate residual IAA. The alkylated Env proteins were buffer‐exchanged into 50 mM Tris/HCl, pH 8.0 using Vivaspin columns (3 kDa) and digested separately overnight using trypsin, chymotrypsin (Mass Spectrometry Grade, Promega), or alpha lytic protease (Sigma Aldrich) at a ratio of 1:30 (w/w). The next day, the peptides were dried and extracted using C18 Zip‐tip (MerckMillipore). The peptides were dried again, resuspended in 0.1% formic acid and analyzed by nanoLC‐ESI MS with an Ultimate 3000 HPLC (Thermo Fisher Scientific) system coupled to an Orbitrap Eclipse mass spectrometer (Thermo Fisher Scientific) using stepped higher energy collision‐induced dissociation (HCD) fragmentation. Peptides were separated using an EasySpray PepMap RSLC C18 column (75 µm × 75 cm). A trapping column (PepMap 100 C18 3 μM, 75 μM × 2 cm) was used in line with the liquid chromatography (LC) before separation with the analytical column. The LC conditions were as follows: 280 min linear gradient consisting of 4%–32% acetonitrile in 0.1% formic acid over 260min followed by 20 min of alternating 76% acetonitrile in 0.1% formic acid and 4% Acn in 0.1% formic acid, used to ensure all the sample had eluted from the column. The flow rate was set to 300 nl/min. The spray voltage was set to 2.5 kV and the temperature of the heated capillary was set to 40°C. The ion transfer tube temperature was set to 275°C. The scan range was 375 − 1500 m/z. Stepped HCD collision energy was set to 15%, 25%, and 45%, and the MS2 for each energy was combined. Precursor and fragment detection were performed using an Orbitrap at a resolution MS1= 120,000, MS2 = 30,000. The AGC target for MS1 was set to standard and injection time set to auto, which involves the system setting the two parameters to maximize sensitivity while maintaining cycle time. Full LC and mass spectrometry (MS) methodology can be extracted from the appropriate Raw file using XCalibur FreeStyle software or upon request.

Glycopeptide fragmentation data were extracted from the raw file using Byos (Version 3.5; Protein Metrics Inc.). The glycopeptide fragmentation data were evaluated manually for each glycopeptide; the peptide was scored as true‐positive when the correct b and y fragment ions were observed along with oxonium ions corresponding to the glycan identified. The MS data was searched using the Protein Metrics 309 *N*‐glycan library with sulfated glycans added manually combined with the Protein metrics 57 Plant *N*‐linked glycan library. The relative amounts of each glycan at each site, as well as the unoccupied proportion were determined by comparing the extracted chromatographic areas for different glycotypes with an identical peptide sequence. All charge states for a single glycopeptide were summed. The precursor mass tolerance was set at 4 and 10 p.p.m. for fragments. A 1% false discovery rate was applied. The relative amounts of each glycan at each site as well as the unoccupied proportion were determined by comparing the extracted ion chromatographic areas for different glycopeptides with an identical peptide sequence. Glycans were categorized according to the composition detected.

HexNAc(2)Hex(10+) was defined as M9Glc, HexNAc(2)Hex(9–4) was classified as oligomannose‐type. Any of these structures containing a fucose were categorized as FM (fucosylated mannose). HexNAc(3)Hex(5‐6)X was classified as Hybrid with HexNAc(3)Hex(5‐6)Fuc(1)X classified as Fhybrid. Complex‐type *N*‐glycans were classified according to the number of HexNAc subunits and the presence or absence of fucosylation. As this fragmentation method does not provide linkage information compositional isomers are grouped, so for example a triantennary glycan contains HexNAc 5 but so does a biantennary glycans with a bisect. Core glycans refer to truncated structures smaller than M3. Any compositions containing a monosaccharide corresponding to a pentose (e.g., Xylose) are classified in the pentose category. Likewise, any glycan composition detected containing at least one fucose or sialic acid were assigned as “Fucose” and “NeuAc,” respectively.

### 2.6 Generating a three‐dimensional (3D) representation of the glycan distribution of recombinant gp140

To generate a 3D representation of the glycan shield of CAP256 Env, SWISS‐MODEL was used. Template search with BLAST and HHblits was performed against the SWISS‐MODEL template library (SMTL, last update: September 15, 2021, last included PDB release: September 10, 2021). The target sequence was searched with BLAST against the primary amino acid sequence contained in the SMTL. A total of 968 templates were found. An initial HHblits profile has been built using the procedure outlined in (Steinegger et al., 2019), followed by 1 iteration of HHblits against Uniclust30 (Mirdita et al., 2017). The obtained profile was then be searched against all profiles of the SMTL. A total of 1120 templates were found. Models are built based on the target‐template alignment using ProMod3 (Studer et al., 2021). Coordinates which are conserved between the target and the template are copied from the template to the model. Insertions and deletions are remodeled using a fragment library. Side chains are then rebuilt. Finally, the geometry of the resulting model is regularized by using a force field. Any modeled ligands were removed, and a representative glycan for each detected glycan composition was added using Coot proximal to the asparagine it is attached to, corresponding to the data generated for CAP256 Env produced in HEK293 cells.

### 2.7 Resolution of gp140 by PAGE

Purified HIV Env gp140 was resolved under native and denaturing conditions using BN‐PAGE and SDS‐PAGE systems, respectively (Margolin et al., 2019). SDS‐PAGE gels were subjected to western blot analysis using polyclonal goat anti‐gp120, as previously reported (van Diepen et al., 2018).

### 2.8 Rabbit immunizations

Animal immunizations were conducted at the Animal Unit of Stellenbosch University in accordance with the guidelines and approval of the Animal Ethics Committee (AEC 019_015). Approximately 3‐month‐old New Zealand White rabbits were randomly assigned to the different experimental groups (*n* = 5 per group). Animals in the protein only experiment were immunized at weeks 0, 4, 12, and 20 with 40 µg of purified protein formulated 1:1 in Alhydrogel® adjuvant. Blood was drawn at weeks 0, 6, and 14 weeks. The experiment was terminated at Week 22. Animals in the prime‐boost experiment were immunized at Weeks 0 and 4 with a bivalent mixture containing 100 µg each of plasmids encoding a Subtype C mosaic Gag and a matched gp150 from CAP256 SU. These vaccines were developed previously (van Diepen et al., 2019) and were administered in CpG ODN 1826 (27.5 µg) adjuvant using the Pharmajet® Stratis (Pharmajet) device. Recombinant MVA encoding the matched antigens was administered using the Pharmajet® Stratis (Pharmajet) device at Weeks 8 and 12 as 10^8^ plaque forming units formulated in 500 µl of posphate‐buffered saline. The construction and characterization of this vaccine was described previously (van Diepen et al., 2019). Finally, animals were boosted with purified protein at Weeks 20 and 28, as described in the protein only arms of the experiment. Blood was drawn at weeks 0, 6, 10, 14, and 22. The experiment was terminated at Week 30. All vaccines were administered intramuscularly by injection into the quadriceps muscle in the hind leg.

### 2.9 Negative‐stain EM

Samples were negatively stained using standard protocols (Booth et al., 2011) and imaged at ×10,000 nominal magnification using a Tecnai T20 TEM (Thermo Fisher). Particles were selected by template‐free auto‐picking using Laplacian‐of‐Gaussian filtering, extracted into 208 × 208 boxes and subjected to two‐dimensional (2D) classification in Relion 3.1 (Scheres, 2012). Nonprotein debris and empty areas of carbon were eliminated during early rounds of classification. The number of images making up the set of 2D classes with approximately the correct size and symmetry were divided by the total number remaining to estimate the proportion of correctly assembled trimeric HIV Env gp140 particles.

### 2.10 Immunogenicity assessment

Sera from immunized rabbits was assessed for binding antibodies by ELISA, as previously described (Margolin et al., 2019) and neutralizing antibodies were quantified using a standardized pseudovirus neutralization assay that was outlined in previous accounts (Margolin et al., 2019; van Diepen et al., 2019)

### 2.11 Statistical analysis

Statistical analyses were conducted using GraphPad Prism 5.0. Comparisons between groups over time were analyzed using a two‐way ANOVA test with a *p* < 0.05 considered as significant.

## 3 DISCUSSION

The development of approaches to engineer glycosylation and folding pathways in plants potentially enables the production of glycoproteins which could not otherwise be viably produced in the system (Margolin, Crispin, et al., 2020; E. A. Margolin et al., 2020). Nonetheless, recapitulating native protein folding and glycosylation still poses a formidable challenge for vaccine development ‐ and viral glycoproteins in particular often accumulate poorly in plants (Margolin et al., 2018). Using soluble HIV Env gp140 as a model for a heavily glycosylated viral glycoprotein, we have systematically investigated bottlenecks that impair the folding and glycosylation of viral glycoproteins in *N. benthamiana* (Margolin et al., 2019, 2021; Margolin, Oh, et al., 2020). In this current study, we have integrated a series of host and engineering approaches to address these limitations and thereby improve the production of the antigen in plants.

Integrating these approaches yielded a dramatic improvement in the production of the recombinant immunogen, which exhibited reduced aggregation and facilitated improved recovery of oligomeric protein. A similar observation was recently described for plant‐produced IgA where the authors reported that coexpression of LmSTT3D improved the formation of dimers in the system by increasing occupancy of PNGs, although the impact was much less dramatic (Goritzer et al., 2020). The GE protein was also better resolved by SDS‐PAGE compared with the non‐engineered progenitor (WT). The basis for the diffuse products that were consistently observed for the WT protein in this and previous work (Margolin et al., 2021) is unclear; however, it is plausible that they arose via aggregation during storage or potentially through inherent instability of the protein. Nonetheless, these observations highlight the critical role of host glycosylation in protein folding and oligomer assembly.

The GE protein contained considerably lower levels of under glycosylated PNGs compared with the WT protein, resulting from coexpression of LmSTT3D, although this was still slightly lower than when the protein was produced in mammalian cells. It is quite remarkable that LmSTT3D could impart this effect in the context of such a heavily glycosylated viral glycoprotein. Previous studies have only tested the coexpression of the enzyme with antibodies and enzymes, which are considerably less glycosylated (Castilho et al., 2018; Goritzer et al., 2020). In stark contrast, the surface glycoprotein of HIV is amongst the most heavily glycosylated proteins described to date and contains ~30 glycans per protomer, which comprises approximately half the mass of the trimer (Stewart‐Jones et al., 2016). This study adds to a growing body of evidence that certain glycoproteins may be less efficiently glycosylated in plants (Castilho et al., 2018; Goritzer et al., 2020; Margolin et al., 2021), although we note that further work is required to determine the extent to which this phenomenon occurs. It is plausible that engineering the protein sequence directly could further improve glycan occupancy and this has recently been reported for mammalian cell‐produced HIV Env trimers (Derking et al., 2021).

The GE protein was decorated almost exclusively with oligomannose‐type glycans, with negligible undesired plant‐specific modifications. The successful production of a recombinant glycoprotein lacking foreign (plant‐specific) glyco‐epitopes is a notable achievement given concerns regarding their potential immunogenicity in humans (Margolin, Crispin, et al., 2020). However, the resulting protein also displayed elevated levels of unprocessed oligomannose‐type glycans compared with the mammalian cell‐produced protein. These are presumed to arise due to inefficient processing by host mannosidases in plants where the extensive glycosylation present on the recombinant protein may have exceeded the capacity of the system to efficiently mediate trimming. This would reduce the formation of complex glycans, providing an explanation as to why the WT protein did not contain plant‐specific complex glycans. It is possible that the use of a weaker promoter driving expression of the HIV Env glycoprotein could improve the efficiency of mannose‐trimming, although this would be counter‐productive where high levels of the target protein are desired for commercial production. This observation may also partly reflect differences in the purification methods for the different systems. In mammalian cells, the protein is secreted into the extracellular media and therefore all of the protein that was purified would have completed its translocation through the secretory pathway. In contrast the plant‐produced gp140 was purified by homogenizing leaves thereby releasing protein from all stages of the secretory pathway. Accordingly, this would enrich for proteins in the early stages of the secretory pathway where glycan maturation would not yet have occurred. Given the fact that the majority of the *N*‐glycans were oligomannose and not processed to complex ones, it is difficult to attribute much impact to the HEXO3RNAi construct used in this study for HIV Env gp140. However, the goal of this study is ultimately to develop a broadly applicable vaccine production platform and this is expected to have a greater impact for other targets with more processed *N*‐glycans

Although no study has reported the site‐specific glycosylation of viral CAP256 glycosylation, it is possible to compare the results obtained in this study with general observations from Env proteins from other strains. Analysis of the BG505 strain revealed two conserved regions of oligomannose‐type glycans, one focused around the N332 supersite, termed the intrinsic mannose patch, and another located at the trimer apex, focused around the N160 site (Behrens et al., 2017; Pritchard et al., 2015; Struwe et al., 2018). These two regions are key bnAb epitopes and the integrity of glycan processing in these areas is key for a successful immunogen. This is true for all immunogens analyzed in this study, with oligomannose‐type glycans present across the outer domain and the apex. Complex‐type glycans are located towards the base of previously analyzed BG505 Env trimers and the mammalian‐derived CAP256 trimer is similarly processed; however, this is not the case for the plant‐derived immunogens. Additionally, under occupancy on gp41 is a common feature of soluble Env immunogens produced in mammalian glycoproteins (Derking et al., 2021) and the presence of the under occupancy on these sites in plant‐derived Env is one that requires solving for all immunogens.

Visualization of the GE and mammalian cell‐produced proteins both failed to yield convincing evidence of native‐like trimer formation. The antigen expressed in both systems was designed (Margolin et al., 2019; van Diepen et al., 2018) using a first generation “native flexible linker” strategy (Sharma et al., 2015), which was subsequently reported to require additional stabilizing mutations to promote efficient trimer formation for some envelopes (Georgiev et al., 2015; Ringe et al., 2015). It is worth noting that the efficiency at which native trimers form is heavily influenced by the genetic background of the virus and Subtype C isolates are often particularly difficult to produce in their native conformation without extensive engineering of the protein (Guenaga et al., 2017; Julien et al., 2015; Ringe et al., 2017). In many cases, homogenous preparations of trimer antigen may also require purification with monoclonal antibodies which specifically capture well‐folded trimers (Ringe et al., 2015) or eliminate misfolded protein species (Guenaga et al., 2015). This complicates vaccine development in Africa, where the infrastructure is poorly developed and preparation of GMP‐grade antibody to support process development would be a further challenge (Margolin, Burgers, et al., 2020). In this study, we deliberately opted not to use mAb‐based purification schemes, but instead opted for sequential affinity chromatography and gel filtration steps that could be widely implemented as would be required for an HIV vaccine.

The promising improvements in glycosylation of the GE gp140 prompted its immunogenicity testing in rabbits, where it was compared with the equivalent mammalian cell‐produced antigen. Binding antibody ELISAs suggested that the plant‐produced antigen yielded slightly lower levels of binding antibodies than the mammalian protein. However, both antigens induced highly similar neutralizing antibodies against both Tier 1 and Tier 2 viruses. Although protein only immunizations did not induce any discernible autologous neutralizing antibodies, priming with DNA and MVA resulted in neutralization of the matched Tier 2 virus following protein boosting. Both plant and mammalian cell‐derived proteins induced similar numbers of responders and comparable neutralizing antibody titers suggesting that the immunogens were similar. However, it is unclear if the antibodies induced by the plant‐produced and mammalian cell‐derived proteins targeted different epitopes. This would not be unexpected given the differences in glycan occupancy that were observed between the proteins and a large body of evidence demonstrating that antibodies frequently target holes in the glycan shield of the HIV Env glycoprotein (McCoy et al., 2016; Wibmer et al., 2016; Yang et al., 2020; Zhou et al., 2017). Therefore, it is plausible that unoccupied sites in the plant‐produced antigen could induce antibodies that would not be induced by the mammalian cell‐produced protein where these epitopes were shielded by glycans. If, however this was the case, this did not appear to negatively impact the induction of neutralizing antibodies.

This study represents a significant advance in plant‐based viral glycoprotein production through engineering of the secretory pathway. These approaches now enable remodeling of the plant factory to better support the maturation requirements of complex glycoproteins facilitating the production of new vaccines. Further work is ongoing to apply this integrated approach to other viral targets, particularly those which pose a threat for causing disease outbreaks. In conclusion, we present a novel paradigm for HIV‐1 Env and other complex glycoprotein production in plants to facilitate rapid, lower cost reagent or vaccine production for developing countries.

## AUTHOR CONTRIBUTIONS

Emmanuel Margolin conceptualized the study with input from Anna‐Lise Williamson, Edward P. Rybicki, Ros Chapman, and Richard Strasser. Emmanuel Margolin and Matthew Verbeek conducted protein expression in plants, whereas protein expression in mammalian cells was conducted by Michiel van Diepen and Phindile Ximba. The site‐specific glycosylation analysis was performed by Joel D. Allen. Animal immunogenicity assays were conducted by Emmanuel Margolin and Thopisang Motlou. Protein expression in plants was overseen by Ann Meyers, Richard Strasser, and Edward P. Rybicki. Protein expression in mammalian cells and animal immunogenicity was supervised by Ros Chapman and Anna‐Lise Williamson. Site‐specific glycosylation analysis was overseen by Max Crispin. Neutralization assays were supervised by Penny L. Moore. Emmanuel Margolin and Joel D. Allen compiled the manuscript. All authors reviewed and approved the final manuscript before submission.

## CONFLICT OF INTERESTS

Emmanuel Margolin, Ann Meyers, Ros Chapman, Anna‐Lise Williamson, Richard Strasser, and Edward P. Rybicki have filed patent applications describing approaches to produce recombinant glycoproteins in plants: WO 2018/069878 A1, WO 2018/220595 A1, PA174002/PCT, PA176498_P.

## Supporting information

Supplementary information.

## Data Availability

Supporting data for the manuscript has been provided in the supplement.
