# Supplementary material for: Augmenting glycosylation‐directed folding pathways enhances the fidelity of HIV Env immunogen production in plants
Source: Biotechnol Bioeng. 2022 Jul 19;119(10):2919–37. doi: 10.1002/bit.28169 (PMC9544252; doi:10.1002/bit.28169)
Supplement: Supplementary file 1 — Supplementary information. [file BIT-119-2919-s001.docx]

Table S1: Site-specific glycan analysis of Env produced in WT *N. benthamiana*

Table S2: Site-specific glycan analysis of Env produced in glyco-engineered *N. benthamiana*

Table S3: Site-specific glycan analysis of Env produced in HEK293 cells

Table S4: Percentage point change in glycan composition and occupancy of Env produced in glyco-engineered *N. benthamiana* compared to WT *N. benthamiana*

Table S5: Percentage point change in glycan composition and occupancy of Env produced in glyco-engineered *N. benthamiana* compared to HEK293 cells
